# Biological interpretation of prognostic radiomic score by correlating with tumor heterogeneity and microenvironment

**DOI:** 10.1186/s13058-022-01527-x

**Published:** 2022-05-09

**Authors:** Jieling Zheng, Bin Zhang

**Affiliations:** Department of Radiology, The First Affiliated Hospital of Jinan University, No. 613 Huangpu West Road, Tianhe District, Guangzhou, 510627 Guangdong China

To the Editor

We read with great interest the article by Dr. Wang and colleagues entitled “radiomics predicts the prognosis of patients with locally advanced breast cancer by reflecting the heterogeneity of tumor cells and the tumor microenvironment,” which was recently published online in *Breast Cancer Research* [1]. To our knowledge, this study is the first attempt to predict individual disease-free survival (DFS) in patients with locally advanced breast cancer who received neoadjuvant chemotherapy and postoperative radiotherapy by radiomics. The authors constructed a radiomic score to predict the prognosis of patients and assessed the associations of radiomic score with the tumor microenvironment and immunophenotype, as well as computational histopathology.

Despite the encouraging results, two issues regarding results need to be clarified. First, the multivariate Cox analysis of clinicopathological factors and radiomic score showed that only age and radiomic score were independent predictors for DFS in the training cohort. The nomogram also displays that the contribution of clinicopathological factors except for age was very small, thus most clinicopathological factors can be discarded. In addition, the range of median DFS probability was very narrow, suggesting the clinical usefulness of the nomogram may be limited. The calibration curves indicate the calibration of the nomogram was not good and the Hosmer–Lemeshow test is necessary to determine the goodness of fit of the nomogram. Second, the C-index of the radiomic nomogram in the training and validation cohorts was 0.820 and 0.612, respectively. Similarly, the C-index of radiomic score alone was 0.810 and 0.614 for training and validation cohorts, respectively. The results of the radiomic score and radiomic nomogram in the two cohorts suggested overfitting.

Radiomic features contain abundant information about tumor biology that is governed not only by cancer cells but also by the tumor microenvironment (TME) [2]. Biological validation of radiomic features remains a critically elusive but important issue for clinical translation of radiomic analysis [3]. In this study, radiomics revealed heterogeneity of tumor cells and tumor microenvironment by gene set enrichment analysis and the assessment of tumor immunophenotype. The biological interpretation of radiomics was mainly via bioinformatic analysis, which was short of adequate experimental evidence. Thus, we are looking forward to furthering investigations for validation. In terms of heterogeneity of tumor cells, GSEA showed that DNA repair, G2/M checkpoint, and PI3K/Akt/mTOR pathways were enriched in both the high- and low-score groups. However, there may be some differential pathways in the two groups, which can reflect the biological differences between these groups and the biological underpinnings of the radiomic score. In terms of heterogeneity of TME, tumor immunophenotype was assessed by MHC molecule score, which was involved with tumor escape mechanisms. However, TME comprises various cell types, including immune cells and stromal cells, cellular factors, and molecules [4]. Tumor immunophenotype can be determined by CD8+ cytotoxic T-cell fractions, immune-suppressive regulatory T cells (T), myeloid-derived suppressor cells, and M2 tumor-associated macrophages (TAMs), which are closely related to the prognosis of breast cancer [5–7]. Hence, tumor immunophenotype should be assessed by more biomarkers with immunohistochemistry, such as M2 TAM (CD68+, CD163+) [8]. Moreover, given that some genes, such as SLURP1, PAX7, UCP1, and ABCA10, were proven to be associated with the prognosis of LABC, and high-score MHC was significantly correlated with molecule score, these merits can be added to the construction of radiomic model to achieve better predictive performance.

## Data Availability

Not applicable.
